# Dietary supplementation of menthol-rich bioactive lipid compounds alters circadian eating behaviour of sheep

**DOI:** 10.1186/s12917-019-2109-0

**Published:** 2019-10-21

**Authors:** Amlan K. Patra, Sebastian Geiger, Hannah-Sophie Braun, Jörg R. Aschenbach

**Affiliations:** 1 Institute of Veterinary Physiology, Freie Universität Berlin, Oertzenweg19b, Berlin, Germany; 2 Department of Animal Nutrition, West Bengal University of Animal and Fishery Sciences, 37 K. B. Sarani, Kolkata, India; 3 PerformaNatGmbH, Hohentwielsteig 6, Berlin, Germany

**Keywords:** Circadian pattern, Eating behaviour, Menthol, Sheep

## Abstract

**Background:**

Plant bioactive lipid compounds (PBLC), commonly known as essential oils, are increasingly evaluated as feed additives in ruminants due to beneficial effects on animal performance and health; however, there is no study evaluating circadian eating behaviour in ruminants. Altered eating behaviour may be implicated in changes of feed intake in ruminants. Therefore, the present study investigated the influence of menthol-rich PBLC on circadian eating behaviour in 24 growing sheep that were equally divided into three treatments, control (without PBLC), a lower dose (80 mg/d) or a higher dose (160 mg/d) of PBLC. Daily doses of PBLC were supplied with 600 g/d of concentrates fed in three equal portions at 07:00, 11:00 and 15:00 h for 4 weeks, whereas, meadow hay was fed ad libitum.

**Results:**

The eating behaviour recorded by an automatic transponder-operated feeding system revealed that daily eating time and feeder visits increased with increasing doses of PBLC. The circadian distribution of eating time and feeder visits (with 1-h resolution) was influenced by the treatment. Eating time during concentrate-offering hours and between concentrate-offering hours increased or tended to increase linearly with greater concentrations of PBLC. Feeder visits did not change significantly during concentrate-offering hours, but were greater in the PBLC groups compared with the control between concentrate-feeding hours. Average length of the longest meals (5th percentile) decreased due to PBLC feeding. Daily feed intake was greater in the PBLC groups than the control.

**Conclusions:**

Menthol-rich PBLC in the applied dose range stimulate circadian eating behaviour, which cannot only be attributed to their presence during concentrate feeding hours, but persist during post-concentrate feeding hours.

## Background

Antibiotic growth promoters have been banned, restricted or placed under scrutiny in several countries of the world including the European Union due to concerns regarding the development of antibiotic resistance to pathogenic bacteria and regarding the presence of antibiotic residues in foods of farm animal origins [1, 2]. This restriction has resulted in a reduction of growth performance of animals and increased prevalence of intestinal diseases, especially in non-ruminant animals [3, 4]. Researchers explored various alternatives to antibiotic growth promoters to cope up with these situations [5–7]. Several plant bioactive compounds including plant bioactive lipid compounds (PBLC; most of them are commonly known as essential oils), flavonoids, tannins and saponins are increasingly evaluated for use as feed additives in farm animals due to the presence of many beneficial properties including antimicrobial, antioxidant, immune-modulating and various other pharmacological activities [5, 8, 9]. In ruminants, some bioactive plant compounds have been shown to modulate ruminal fermentation and improve production performance and health status of livestock while offering environmental advantages in few studies [8, 10–12].

Feed intake, feed preference and eating behaviour of ruminants are regulated by multiple interacting determinants, including physiological (e.g., leptin, adiponectin, ghrelin, insulin, glucocortocoid hormones and neuropeptide Y/Agouti-related protein [13, 14]), nutritional (e.g., feed characteristics, digestive fill and energy requirement [15, 16]), environmental (e.g., temperature and duration and intensity of light [17]) and social factors. Among these factors, feed characteristics are important contributors to eating behaviour in ruminants and comprise mainly physical structure (particle size, shear resistance, height and density of pasture sward; determining touch sensory responses and ease of prehension and mastication) and chemical properties (energy density, taste and smell) [15, 16]. Many PBLC may exert distinctive olfactory, gustatory and trigeminal nerve-mediated chemosensory stimuli [18]. Thereby, they may potentially alter eating behaviour of animals depending upon the type and amount present in feeds [19, 20]. A few studies in ruminants showed that feeding of PBLC indeed changed total eating time and meal size and length [6, 21]. Nonetheless, there are limited studies that investigated the effects of PBLC on eating behaviour monitored over 24 h of day and a prolonged period of time.

Menthol or menthol-containing plants and oils exhibit many beneficial biological activities and they have been tested to modulate rumen microbial fermentation and feed utilisation [22, 23]. Menthol also activates bovine transient receptor potential (TRP) channels in the ruminal epithelium and stimulates cation absorption [24, 25]. Furthermore, menthol has potent trigeminal and other chemosensory stimulant properties in the oral and nasal mucosa mediated via TRP channels [26, 27]. Based on the distinctive chemosensory properties of menthol, we hypothesised that menthol-containing PBLC could alter the eating behavioural activities mediated by associations between sensory properties and post-ingestinal feedback. As chemosensation may elicit dose-dependent effects that may, e.g., include attraction at lower doses and aversion at higher doses, the dose effects were also of interest. Therefore, an experiment was conducted to study the effect of two linearly increased dosages of dietary PBLC with menthol as a main compound on eating behaviour of sheep.

## Results

A summary of the least square mean data over the whole observation period of 3 weeks is presented in Table 1; whereas, data for individual weeks or time points are presented in the figures and also as additional information.

**Table 1:** Effect of two doses of dietary menthol-rich plant bioactive lipid compounds (PBLC) on eating behaviour of sheep

Attribute	Treatment (*n* = 8)^a^			SEM^b^	*P* values^c^			
	Control	PBLC-L	PBLC-H		Treatment	L	Q	C vs. PBLC
Eating time (min/d)^d^	264^x^	263^x^	290^y^	8.6	0.034	0.046	0.19	0.25
Feeder visit (number/d)^d^	228^x^	268^xy^	288^y^	18.0	0.047	0.028	0.68	0.036
Average meal length (min)^e^								
Upper 5th percentile	6.01^x^	5.05^y^	5.27^xy^	0.324	0.048	0.12	0.16	0.047
Upper 10th percentile	5.73	4.91	5.03	0.307	0.16			0.060
Upper 25th percentile	4.76	4.37	4.37	0.232	0.41			0.19
Eating rate (g/min)^f^	4.72	4.85	4.49	0.155	0.26			0.80

^x,y^Treatment means with different superscripts differ significantly (*P* < 0.05) within a row

^a^Least square mean values are reported for treatments: control, without PBLC; PBLC-L, lower dose (80 mg/d) of PBLC; and PBLC-H, higher dose (160 mg/d) of PBLC

^b^Standard error of mean

^c^Contrast: *L* Linear effect, *Q* Quadratic effect, and *C vs. PBLC* Control versus pooled PBLC groups

^d^Average values of last 3-week data

^e^Meal length was determined in the last week

^f^Week (*P* = 0.61) and week × treatment (*P* = 0.80) effects were not significant

### Weekly eating behaviour

#### Eating time

**Fig. 1:**
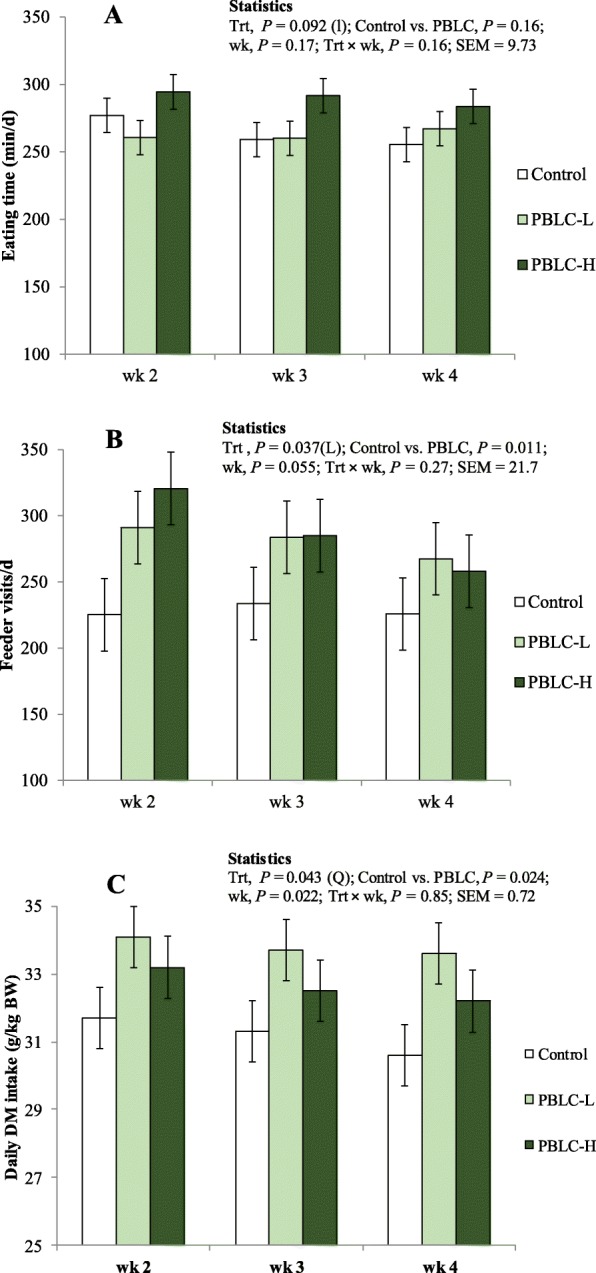
Effect of different doses of menthol-rich plant bioactive lipid compounds (PBLC) on **a** eating time, **b** frequency of feeder visits and **c** daily intake of feed dry matter (DM) in different weeks of feeding. Sheep (*n* = 8 per treatment) were fed diets containing 0 mg/d (control), 80 mg/d (PBLC-L) and 160 mg/d of PBLC (PBLC-H), respectively. Trt, treatment; wk., week; DM, dry matter; BW, body weight; L, significant (*P* < 0.05) linear, and l and q, trend (*P* < 0.10) for linear and quadratic effects of PBLC dose; SEM, standard error of mean

Average daily eating time during the last 3 weeks of the 4-week period increased linearly (*P* = 0.046) with increasing PBLC dose and was greater for PBLC-H versus control (Table 1). The weekly analysis also showed that daily eating time tended to increase linearly (*P* = 0.092) with increasing concentrations of PBLC in the diets, but was not affected by week (*P* = 0.17) and treatment × week interaction (*P* = 0.16; Fig. 1a and Additional file 1: Table S1).

#### Feeder visits

Average daily eating frequency also increased linearly (*P* = 0.028) with increasing PBLC dose during the 3-weeks period and was higher when the pooled PBLC-supplemented groups were compared to the control group (*P* = 0.036; Table 1). Accordingly, weekly analysis of eating frequency showed that daily feeder visits by sheep increased linearly with greater levels of PBLC (*P* = 0.037; Fig. 1b, Additional file 1: Table S1). Eating frequency tended to decrease over time from week 2 to week 4 (*P* = 0.055), but was not affected (*P* = 0.27) by treatment × week interaction (Fig. 1b, Additional file 1: Table S1).

### Circadian eating behaviour

#### Eating time

**Fig. 2:**
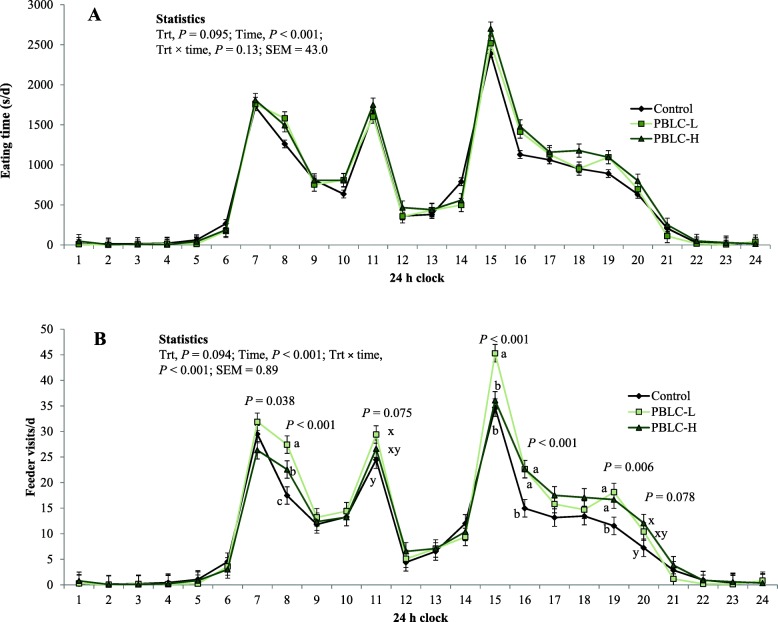
Effect of different doses of menthol-rich plant bioactive lipid compounds (PBLC) on **a** the circadian distribution of eating time and **b** feeder visits in sheep. Sheep (*n* = 8 per treatment) were fed diets containing 0 mg/d (control), 80 mg/d (PBLC-L) and 160 mg/d of PBLC (PBLC-H), respectively. Concentrates were fed at 07:00, 11:00 and 15:00 h of the day. Because interaction effect for feeder visits was significant, ‘slice’ option in the SAS mixed model was used to detect the significant difference at a time point and subsequently mixed model procedures were employed to analyse the treatment effect at that particular time point using Fisher’s protected least square difference test. ^a-c^Means followed by different letters within a time point differ at *P* < 0.05. ^x-y^Means followed by different letters within a time point differ at *P* < 0.10. Trt, treatment; SEM, standard error of mean

**Fig. 3:**
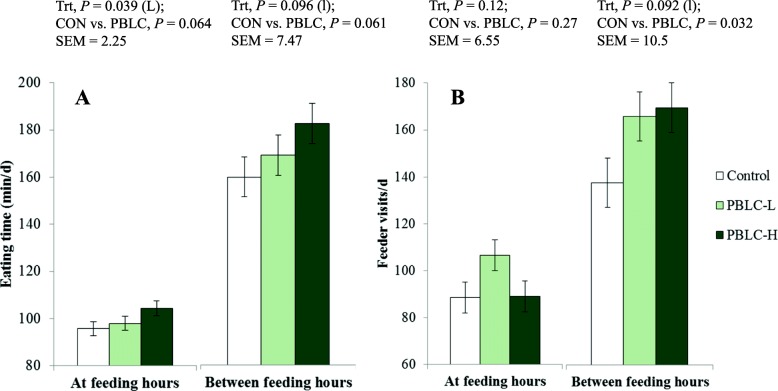
Effects of different doses of menthol-rich plant bioactive lipid compounds (PBLC) on **a** eating time and **b** frequency of feeder visits during and between feeding hours in sheep. Sheep (*n* = 8 per treatment) were fed diets containing 0 mg/d (control, CON), 80 mg/d (PBLC-L) and 160 mg/d of PBLC (PBLC-H), respectively. Trt, treatment; L, significant (*P* < 0.05) linear effect; l, trend (*P* < 0.10) in linear effect; and q, trend (*P* < 0.10) in quadratic effect of PBLC dose; SEM, standard error of mean

In week 4, hourly eating time (min/h) showed circadian variation (*P* < 0.001) and tended to be affected by the diet (*P* = 0.095), but the interaction effect between treatment × hour-of-day was not significant (*P* = 0.13; Fig. 2a, Additional file 2: Table S2). Accordingly, eating time increased with greater concentrations of PBLC both during concentrate feeding hours (07:00, 11:00 and 15:00 h; *P* = 0.039 with a significant linear response) and between concentrate feeding hours (i.e., the remaining 21 h; *P* = 0.096 with a tendency for linear response; Fig. 3a, Additional file 3: Table S3).

#### Feeder visits

Circadian distribution of eating frequency was affected by the treatment × hour-of-day interaction (*P* < 0.001) during the 24 h clock period (Fig. 2b, Additional file 2: Table S2). For PBLC-L, eating frequency was greater (*P* < 0.05) at 08:00 h, 15:00 h, 16:00 h and 19:00 h, and tended to be greater (*P* < 0.10) at 11:00 h compared with the control diet (Fig. 2b). For PBLC-H, eating frequency was greater (*P* < 0.05) at 08:00 h, 16:00 h, and 19:00 h, and tended to be greater (*P* < 0.10) at 20:00 h compared with the control diet (Fig. 2b). Overall, eating frequency during concentrate offering hours did not change (*P* = 0.12), but was numerically greater for PBLC-L (Fig. 3b, Additional file 3: Table S3). The eating frequency in other hours tended to increase linearly (*P* = 0.092) with increasing concentration of PBLC in the diets and it was higher (*P* = 0.032) for pooled PBLC groups versus control.

### Feed intake, meal length, eating rate, and body weight gain

Concentrate intake was similar among groups as all the groups were given equal amounts of concentrate and sheep consumed concentrates immediately and completely. Daily feed intake was not affected (*P* = 0.85) by the treatment × week interaction, but was affected by treatment (*P* = 0.043) in a quadratic manner (*P* < 0.037) and with greater values (*P* = 0.024) for the pooled PBLC groups versus the control group. Daily feed intake was further affected by week (*P* = 0.024; Fig. 1c, Additional file 4: Table S4). Although the upper quartile (i.e., 25th percentile) of meal length was similar (*P* = 0.41) among treatments, averages of top 10th (*P* = 0.060) and 5th (*P* = 0.047) percentiles of meal length were lower for the pooled PBLC groups than the control group (Table 1). Eating rate (in g/min) by sheep was not affected by treatment (*P* = 0.26), week (*P* = 0.61) and treatment × week interaction (*P* = 0.80). In each experimental group, significant positive correlations existed between daily DM intake and daily eating time, i.e., *r* = 0.71 (*P* = 0.050; *n* = 8), 0.80 (*P* = 0.029; *n* = 7) and 0.73 (*P* = 0.040; *n* = 8) for control, PBLC-L and PBLC-H, respectively (Additional file 6: Figure S1). By contrast, no correlation existed between eating rate and meal length (*r* = − 0.48, 0.38 and − 0.47 for control, PBLC-L and PBLC-H, respectively; *P* > 0.10 each). Body weight gain over the four experimental weeks averaged to 236 ± 13.1 g/d with no difference among groups (*P* = 0.82; Additional file 3: Table S3).

## Discussion

Circadian rhythms are present in almost all living organisms. They are controlled by endogenous autonomous oscillators that approximate biological functions at the molecular, physiological and behavioural level to the 24-h day. Apart from light-dark cycle, feeding-fasting patterns are amongst the most important external cues that influence the robustness of daily biological rhythms [28]. The latter became evident in the present experimental study. Despite hay was constantly available to the animals, they adapted their eating behaviour to the timed provision of the more palatable concentrates. In addition, the supplementation of menthol-rich PBLC to the concentrates further impacted on eating behaviour. Because sheep were all kept in the same barn and fed concentrates at the same time, we can exclude that the PBLC effect on eating behaviour was a procedural artefact caused by the process of feed provision.

Menthol is a potent trigeminal and other chemosensory stimulant in the oral and nasal mucosa mediated via TRP channels in a dose-dependent manner [26, 27]. Thus, we hypothesised that menthol-containing PBLC could modulate the eating behavioural activities dose-dependently. In the present study, daily eating time increased due to PBLC feeding in sheep, and this effect persisted for 4 weeks. In previous experiments, the effects of in-feed PBLC on intake time were conflicting depending upon the type and dose of PBLC used. For example, supplementation of copaiba PBLC (0.5 to 1.5 g/kg DM) linearly increased eating time without affecting DM intake in lambs [29]. By contrast, cinnamaldehyde (3.5 and 7 g/d) or clove oil (3.5 and 7 g/d) had no influence on eating time in steers although feed intake increased linearly [11]. A mixture of cinnamaldehyde and eugenol (up to 1.7 + 2.8 g/d) or capsicum oil (0.25 g/d) did also not influence eating time in lactating dairy cows [21]. A PBLC mixture (300 mg/d; containing 430 g/kg of cinnamaldehyde and 70 g/kg of garlic oil) added to a total mixed ration of lactating dairy cows decreased eating time slightly (172 versus 164 min/d) without affecting feed intake [6].

There are no studies showing the effects of PBLC on visits to the feeders and circadian eating patterns in ruminant animals. We assumed that greater intake time should result most likely from more frequent feeder visits by sheep. Hedonic preferences of feeds influence motivation to visit feeders to consume more feeds [15]. Surprisingly, we noted more frequent feeder visits not only during concentrate offering hours but also after concentrate offering hours in the present study. One simple explanation for the stimulation of feed intake after concentrate offering hours could be the presence of PBLC in digesta reaching the oral cavity during rumination. On the other hand, PBLC may also stimulate feed intake for certain periods after their actual presence. In a human study, olfactory and gustatory sensations continued over the time course of a meal for a given food without actual food intake [30]. Menthol is an effective agonist of TRP (e.g., TRPM8) receptors [26, 27] that are potentially involved in feed intake and energy metabolism [31]. Stimulation of the TRPM8 channel by menthol generates a sensation of cooling and, in some studies, this has been shown to influence thirst, arousal and food intake in humans [32]. Therefore, it seems possible that menthol-containing PBLC altered hunger drive and initiation of eating by either directly modulating TRP channel activities or by indirectly affecting other behavioural modes that fed forward on feed intake.

Though daily eating time increased due to PBLC feeding, the top 5th and 10th percentile meal length average was or tended to be shorter in the pooled PBLC groups than in the control group. Meal size or length is regulated by internal mechanisms relating to short-term post-ingestive triggers (e.g., rumen load, toxicity and nutritive value) that control nutritional excess (satiation effect) and avoid undesirable toxic principles in the body [33]. In a previous short-term (6 d) study, sheep fed a complete concentrate diet containing different PBLC (eucalyptus, mint, orange and oregano oils at 5 ml/kg diet) on a free-choice basis had shorter meal length and decreased feed intake initially compared with sheep fed the same concentrate without any PBLC [20]. This effect was interpreted as neophobia to diets containing atypical components; as a mechanism to restrict entry of undesirable compounds into the body [20, 34]. Pre-ingestive characteristics (i.e., texture, olfactory and gustatory properties) of feeds are contributing factors affecting palatability and hedonic values of diets and influencing feed intake [20, 33]. Feed neophobia likely occurs when an unfamiliar feed or flavour is introduced in diets of sheep [34] and behavioural mechanisms develop to recognise and select feeds on the basis of nutritional properties and previous experience of feed consumption [18, 35], which modify their intake and preferences. In a taste preference study with different doses of cinnamaldehyde in diets (0 to 4 mg/kg body weight), dairy heifers preferred diets without cinnamaldehyde, but did not reduce feed intake when control diets were not available regardless of cinnamaldehyde concentrations in the diets [36]. However, sheep of the present study consumed PBLC concentrates quickly from the first day in equal time as the control sheep although no sheep consumed the PBLC previously. Thus, the generally shorter duration of the 5% longest meals after PBLC-supplementation was not likely attributed to neophobia or to adverse toxic risks by sheep.

Rumen fill is another factor limiting meal size or length [33]. Increased rumen fill could, for example, result from decreased digestion and turnover rates of digesta in the rumen [37]. However, PBLC was used in two comparatively low doses in the present experiment to avoid adverse effects on ruminal digestion. Moreover, eating rate was unaffected by the treatments, suggesting apparently no influence of rumen load on meal length. Therefore, it is likely that metabolic changes in the body caused by PBLC motivated sheep to visit feeders more frequently with post-ingestive nutritional restriction triggering an earlier termination of the longest meals. In lactating dairy cattle, feeding of a mixture of cinnamaldehyde (1.7 g/d) and eugenol (2.8 g/d) or capsicum oil (0.25 g/d) did not affect mean meal length, but reduced the length of the first meal [21].

Daily feed intake tended to increase quadratically by PBLC treatments without any treatment × week interaction. As the amount of concentrate was fixed, the trend for increased feed intake was solely due to increased intake of hay. The trend for an increase in feed intake was not followed by a similar trend for increased body weight gain. The latter will not be discussed any further because it was not the primary focus of this study and it may be attributable to factors related or not related to PBLC (e.g. varying rumen fill during weighing).

The quadratic response of feed intake may suggest that the lower dose of PBLC in this study was better suited to stimulate feed intake than the higher dose. In previous studies, feeding of PBLC showed inconsistent results with similar-to-control feed intake (0.5 and 10 g/d of a mixture of cinnamaldehyde and eugenol and 0.25 g/d of capsicum oil in lactating Holstein cows [21], copaiba PBLC at 0.5 to 1.5 g/kg DM in sheep [29]), greater feed intake (cinnamaldehyde at 0.4 to 1.6 g/d in steers [38], rosemary PBLC at 0.5 g/kg concentrate in sheep [39], 3.5 and 7 g/d of clove or cinnamon oil in bulls [11]) or even lower feed intake (PBLC mixture at 1.2 g/d in lactating dairy cows [40]) depending upon dose and type of PBLC [19]. It is unlikely that the process of food delivery had any contribution to the stimulation of feed intake by PBLC because sheep were all kept in the same barn and fed simultaneously at the same time. The altered feed intake might finally result from increased feed digestion and absorption rates due to alterations of rumen microbial fermentation and metabolic changes in the body. The latter reason has not been studied in ruminants, but PBLC may change metabolic and hormonal profiles [41, 42] that are associated with energy metabolism and hunger [16].

From the above discussions, it can be stated that altered eating behaviour patterns are commonly attributed to the hedonic properties of the used PBLC; however, they may also result from postprandial changes in feed intake regulation. In future studies, it would be interesting to investigate if the circadian change in eating pattern by PBLC might have resulted from alterations in the secretion of hormones and/or neuropeptides that regulate feed intake.

## Conclusions

Feeding of menthol-rich PBLC changed eating patterns (i.e., eating time, meal length and frequency of eating) in sheep, which persisted over the whole duration of the study. Circadian eating behaviour of animals was also altered by diets containing PBLC, which included a drive for more frequent feeder visits in the hours following the actual ingestion of the PBLC.

## Methods

This study was part of a larger trial in which several further readouts were determined after the end of the present trial (e.g., composition of ruminal microbiota [43], transport and barrier function of gastrointestinal epithelia, gene expression and composition of tissue samples). Therefore, although no animal was specifically killed for the present study, all sheep were humanely slaughtered in the week following this trial. Slaughter was done by penetrative captive bolt with subsequent exsanguination by bleeding of jugular veins and was covered by the ethics approval mentioned in the Declarations section.

### Experimental design and animals

Twenty-four growing Suffolk sheep (15 females and 9 males) with no prior PBLC supplementation were purchased from a local farmer. They were kept in quarantine and adapted to a control diet for at least 2 weeks before allocating them to different diets.

The initial body weight and age of the animals were 32.9 ± 3.44 kg and 121 ± 3.7 d, respectively when the experiment started. The experiment was conducted in two runs with 12 sheep in each run and sheep were equally divided into three dietary groups in a randomised block design based on initial body weight (considered as block) and sex (5 females and 3 males in each treatment). There were four blocks with each block containing one sheep of similar body weight from each dietary group; i.e., there were a total of three sheep in each block per run. The three groups were fed a diet either without PBLC (control), with a lower dose (80 mg/d) of PBLC (PBLC-L; OAX17, PerformaNat GmbH, Germany) or with a higher dose (160 mg/d) of PBLC (PBLC-H).

**Fig. 4:**
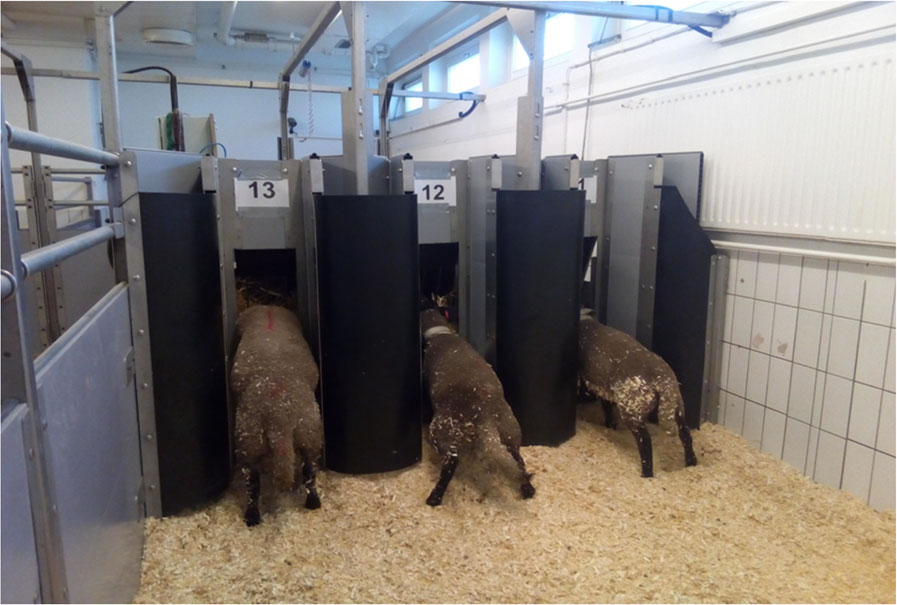
Layout of feeders with transponder-operated feeding stations and pneumatically operated locking gates. A gate opens exclusively when a sheep fitted with an identification tag (transponder) to its neck collar comes close to the feeder with complementary receiver. A sheep can consume feeds only from its allocated feeder

The four blocks of sheep in each run were kept in the four available pens with each pen containing three separate feeders (Fig. 4). Thus, each pen accommodated three sheep with their allotted treatments. Sheep pens had concrete floors with wood shavings as bedding materials. The barn had artificial light that was automatically switched on at 07:00 h and switched off at 18:00 h to support the natural daylight available from glass windows. The experiment was conducted in the months of June to August, 2017. Each sheep was allowed access to one transponder-operated feeding station with locking gate (Hütter GbR, Marktbergel, Germany) that recognised only this individual sheep by animal identification tag (transponder) fitted to its neck collar. Initially, sheep were trained for 2 to 4 d to get them adapted to the automatic feeding system. Both hay and concentrate were offered using the automatic feeding system. All sheep quickly learned to recognise their own feeder.

### Feeding

All sheep were fed the pelleted control concentrate (400 g/d) and ad libitum meadow hay (without chopping) for 4 d during an adjustment period to the automatic feeding system that preceded the experiment. Thereafter, they were provided three different concentrates in containers designed for concentrated feeds, and the amounts of concentrates were gradually (450 g/d for first 3 d and 525 g/d for next 3 d in 3 equal portions) increased to 600 g/d for the adaptation to the experimental concentrated feeds. Hay was provided in the forage storage containers of the feeding stations for ad libitum intake. Ingredients and chemical composition of the three concentrates (organic matter, 949 g/kg; crude protein, 259 g/kg and neutral detergent fibre, 139 g/kg on dry matter (DM; 914 g/kg) basis) were identical except that concentrates of the PBLC-L and PBLC-H groups were supplemented with PBLC at 133.3 and 267.6 mg/kg concentrate (Additional file 5: Table S5). The PBLC contained menthol (90%) with other minor bioactive compounds. Chemical composition of hay was as follows: organic matter, 958 g/kg; crude protein, 108 g/kg and neutral detergent fibre, 641 g/kg on DM basis*.* Hay and concentrate feeds were sampled weekly. Chemical composition of DM, organic matter, crude protein and neutral detergent fibre concentrations in feed was determined following standard methods [44]. Ad libitum hay plus 600 g/d pelleted concentrate diets were fed to meet nutrient requirements for a growth rate of 300 g/d [45]. Water was freely available at all times from push-button water troughs (Modell 370, Suevia Haiges GmbH, Kirchheim, Germany). During initial adaptation period, water buckets were also provided in each pen, but sheep quickly adapted to drink water from the push-button water troughs. The daily dose of PBLC was supplied with the concentrate pellets that were provided in three equal portions (200 g each feeding) at 07:00, 11:00 and 15:00 h. Within 2 to 3 min, concentrates were delivered to all sheep into their allocated concentrate containers of the feeding troughs. Concentrate mixtures containing PBLC had been pelleted below 50 °C to prevent loss of PBLC during pelleting process, and the concentrate pellets were stored in air-tight bags. After 1 week of adaptation to diets, feed consumption by each sheep was recorded weekly for 3 weeks. No orts remained from the concentrates. Orts from hay (usually hard and long stems) were collected daily in a polythene bag for each sheep and pooled over a week. Feed intake was calculated weekly by subtracting the orts left from the amount of feed offered.

### Eating behaviour

After 1 week of adaptation to diets, eating behaviour data were recorded for 3 weeks using the computed records of the automatic feeding system. Each time an animal approached its allocated feeding station, the transponder communicated with the self-designed computer software via a receiver to open the pneumatically driven locking gate. After the animal left the station and the transponder moved outside the receiver’s reception radius, the locking gate immediately closed. The process computer recorded the date, time and duration when a gate opened, as well as if the gate was closed or open. Eating time was determined based on the total time a gate remained open. Number of visits to a feeder was determined from the number of times of a gate opened and closed. To exclude bias by exploration behaviour (i.e., by unintentional approaches to the feeder or by ‘playing’ with the locker gates), a feeder visit of 10 s or less was not considered an actual visit for eating purpose and was excluded from the determination of eating pattern. Meal length was defined as the total time a gate remained opened during an event (or a visit to feeder). To assess whether the treatments would animate the animals to have long meals, the top 5th, 10th and 25th percentile of meal length were determined over the last week. Data were stored by the process computer and were retrieved and exported to excel sheets at the end of each run. Data on eating time and feeder visits were calculated on a weekly basis for each animal during week 2, week 3 and week 4. During the week 4, data of eating time and feeder visits were additionally analysed at hourly resolution for each animal to determine the circadian eating profile. Eating rate (g/min) of each sheep was calculated by dividing daily DM intake by daily eating time.

### Statistical analyses

Outliers (> or < median ± 2.5 median absolute deviation) for all variables, if any, were checked and removed before statistical analyses [46]. Data were analysed using PROC MIXED procedures of SAS [47]. The data were checked for normal distribution. The model included treatment, block, experimental run, sex, treatment × week or treatment × hour-of-day with repeated measure models for the data in week or hour-of-day with animal as a random effect and using variance compound or compound symmetry structure that resulted in better model fit. If effects of block, sex or experimental run were not significant (*P* > 0.05) or increased *P*-values, they were removed before final analysis. When interaction effect was significant, ‘slice’ option in the SAS was used to detect the significant difference in a time point and subsequently mixed model procedures were employed to analyse the treatment effect in that particular time point using Fisher’s protected least square difference test. The mixed model for analysis of 3-week mean data (eating time and feeder visit) and meal length contained treatment, block, run and sex. For treatment *P*-values ≤0.10, linear and quadratic effects of PBLC doses (0, 80 and 160 mg/d) were assessed using polynomial contrasts. Contrasts between control (0 mg/d of PBLC) versus the pooled PBLC groups (80 and 160 mg/d of PBLC) were also used to determine the overall effects of PBLC compared with the control. Pearson correlation coefficient (*r*) between daily DM intake and eating time was analysed using the PROC CORR procedure of SAS [47]. Variability in the data was expressed as the pooled SEM, and statistical significance was set at *P* ≤ 0.05, while a trend was considered at 0.05 < *P* ≤ 0.10.

## Supplementary information

**Additional file 1: Table S1.** Effect of different doses of menthol-rich plant bioactive lipid compounds (PBLC) on eating time and frequency of feeder visits in different weeks of feeding.

**Additional file 2: Table S2.** Effect of different doses of menthol-rich plant bioactive lipid compounds (PBLC) on the circadian distribution of eating time and feeder visits in sheep.

**Additional file 3: Table S3.** Effects of different doses of menthol-rich plant bioactive lipid compounds (PBLC) on eating time and frequency of feeder visits during and between feeding hours, and on body weight gain in sheep.

**Additional file 4: Table S4.** Effect of different doses of menthol-rich plant bioactive lipid compounds (PBLC) on daily intake (g/kg body weight) of feed dry matter (DM) in different weeks.

**Additional file 5: Table S5.** Ingredient and chemical composition of pelleted concentrates and hay fed to sheep.

**Additional file 6: Figure S1.** Correlations between daily feed DM intake and daily eating time in different treatment groups. A = control, B = PBLC lower dose (80 mg/d) and C = PBLC higher dose (160 mg/d).

## Data Availability

The datasets supporting the conclusions of this article are included within the article and its additional files.

## Abbreviations

BW: Body weight
DM: Dry matter
PBLC: Plant bioactive lipid compound
PBLC-H: Group supplemented with 160 mg/d PBLC
PBLC-L: Group supplemented with 80 mg/d PBLC
SEM: Standard error of means
TRP: Transient receptor potential

## References

[1] Woolhouse M, Ward M, van Bunnik B, Farrar J (2015). Antimicrobial resistance in humans, livestock and the wider environment. Phil Trans R Soc.

[2] Brown K, Uwiera RRE, Kalmokoff ML, Brooks SPJ, Inglis GD (2017). Antimicrobial growth promoter use in livestock: a requirement to understand their modes of action to develop effective alternatives. Int J Antimicrob Agents.

[3] Wierup M (2001). The Swedish experience of the 1986 year ban of antimicrobial growth promoters, with special reference to animal health, disease prevention, productivity, and usage of antimicrobials. Microb Drug Resist.

[4] Dibner JJ, Richards JD (2005). Antibiotic growth promoters in agriculture: history and mode of action. Poult Sci.

[5] Huyghebaert G, Ducatelle R, Van Immerseel F (2011). An update on alternatives to antimicrobial growth promoters for broilers. Vet J.

[6] Blanch M, Carro MD, Ranilla MJ, Viso A, Vázquez-Anón M, Bach A (2016). Influence of a mixture of cinnamaldehyde and garlic oil on rumen fermentation, feeding behavior and performance of lactating dairy cows. Anim Feed Sci Technol.

[7] Kumar P, Patra AK, Mandal GP, Samanta I, Pradhan S (2017). Effect of black cumin seeds on growth performance, nutrient utilization, immunity, gut health and nitrogen excretion in broiler chickens. J Sci Food Agric.

[8] Patra AK, Saxena J (2010). A new perspective on the use of plant secondary metabolites to inhibit methanogenesis in the rumen. Phytochemistry.

[9] Chowdhury S, Mandal GP, Patra AK, Kumar P, Samanta AK (2018). Different essential oils in diets of broiler chickens: 2. Gut microbes and morphology, immune response, and some blood profile and antioxidant enzymes. Anim Feed Sci Technol.

[10] Mirzaei-Alamouti H, Moradi S, Shahalizadeh Z, Razavian M, Amanlou H, Harakinejad T (2016). Both monensin and plant extract alter ruminal fermentation in sheep but only monensin affects the expression of genes involved in acid-base transport of the ruminal epithelium. Anim Feed Sci Tech.

[11] Ornaghi MG, Passetti RAC, Torrecilhas JA, Mottin C, Prado IN (2017). Essential oils in the diet of young bulls: effect on animal performance, digestibility, temperament, feeding behavior and carcass characteristics. Anim Feed Sci Technol.

[12] Soltan YA, Natel AS, Araujo RC, Morsy AS, Abdalla AL (2018). Progressive adaptation of sheep to a microencapsulated blend of essential oils: ruminal fermentation, methane emission, nutrient digestibility, and microbial protein synthesis. Anim Feed Sci Technol.

[13] Trellakis S, Tagay S, Fischer C, Rydleuskaya A, Scherag A, Bruderek K (2011). Ghrelin, leptin and adiponectin as possible predictors of the hedonic value of odors. Regul Pept.

[14] Maniam J, Morris MJ (2012). The link between stress and feeding behavior. Neuropharmacology.

[15] Baumont R (1996). Palatability and feeding behavior in ruminants - a review. Ann Zootech.

[16] Ginane C, Bonnet M, Baumont R, Revell DK (2015). Feeding behaviour in ruminants: a consequence of interactions between a reward system and the regulation of metabolic homeostasis. Anim Prod Sci.

[17] De K, Kumar D, Saxena VK, Thirumurugan P, Naqvi SMK (2017). Effect of high ambient temperature on behavior of sheep under semi-arid tropical environment. Int J Biometeorol.

[18] Simitzis PE, Deligeorgis SG, Bizelis JA, Fegeros K (2008). Feeding preferences in lambs influenced by prenatal flavour exposure. Physiol Behav.

[19] Estell RE, Fredrickson EL, Anderson DM, Havstad KM, Remmenga MD (2002). Effects of four mono- and sesquiterpenes on the consumption of alfalfa pellets by sheep. J Anim Sci.

[20] Simitzis PE, Feggeros K, Bizelis JA, Deligeorgis SG (2005). Behavioural reaction to essential oils dietary supplementation in sheep. Biotechnol Anim Husb.

[21] Tager LR, Krause KM (2011). Effects of essential oils on rumen fermentation, milk production, and feeding behavior in lactating dairy cows. J Dairy Sci.

[22] Ando S, Nishida T, Ishida M, Hosoda K, Bayaru E (2003). Effect of peppermint feeding on the digestibility, ruminal fermentation and protozoa. Livest Prod Sci.

[23] Patra AK, Yu Z (2012). Effects of essential oils on methane production and fermentation by, and abundance and diversity of, rumen microbial populations. Appl Environ Microbiol.

[24] Rosendahl J, Braun HS, Schrapers KT, Martens H, Stumpff F (2016). Evidence for the functional involvement of members of the TRP channel family in the uptake of Na^+^ and NH_4_^+^ by the ruminal epithelium. Pflugers Arch.

[25] Schrapers KT, Sponder G, Liebe F, Liebe H, Stumpff F (2018). The bovine TRPV3 as a pathway for the uptake of Na^+^, Ca^2+^ and NH^4+^. PLoS One.

[26] Karashima Y, Damann N, Prenen J, Talavera K, Segal A, Voets T (2007). Bimodal action of menthol on the transient receptor potential channel TRPA1. J Neurosci.

[27] Gerhold KA, Bautista DM (2009). Molecular and cellular mechanisms of trigeminal chemosensation. Ann N Y Acad Sci.

[28] Manoogiana ENC, Panda S (2017). Circadian rhythms, time-restricted feeding, and healthy aging. Ageing Res Rev.

[29] Moura LV, Oliveira ER, Fernandes ARM, Gabriel AMA, Silva LHX, Takiya CS (2017). Feed efficiency and carcass traits of feedlot lambs supplemented either monensin or increasing doses of copaiba (*Copaifera spp*.) essential oil. Anim Feed Sci Technol.

[30] Rolls ET, Rolls JH (1997). Olfactory sensory-specific satiety in humans. Physiol Behav.

[31] Ahern GP (2013). Transient receptor potential channels and energy homeostasis. Trends Endocrinol Metab.

[32] Hutchings SC, Horner KM, Dible VA, Grigor JM, O’Riordan D (2017). Modification of aftertaste with a menthol mouthwash reduces food wanting, liking, and ad libitum intake of potato crisps. Appetite.

[33] Favreau A, Ginane C, Baumont R (2010). Feeding behaviour of sheep fed lucerne v. grass hays with controlled post-ingestive consequences. Animal.

[34] Provenza FD, Lynch JJ, Cheney CD (1995). Effects of a flavor and food restriction on the response of sheep to novel foods. Appl Anim Behav Sci.

[35] Wang J, Provenza FD (1997). Dynamics of preference by sheep offered foods varying in flavors, nutrients, and a toxin. J Chem Ecol.

[36] Chapman CE, Cabral RG, Aragona KM, Erickson PS (2016). Short communication: Cinnamaldehyde taste preferences of weaned dairy heifers. J Dairy Sci.

[37] Dado RG, Allen MS (1995). Intake limitations, feeding behavior, and rumen function of cows challenged with rumen fill from dietary fiber or inert bulk. J Dairy Sci.

[38] Yang WZ, Ametaj BN, Benchaar C, He ML, Beauchemin KA (2010). Cinnamaldehyde in feedlot cattle diets: intake, growth performance, carcass characteristics, and blood metabolites. J Anim Sci.

[39] Smeti S, Joy M, Hajji H, Alabart JL, Muñoz F, Mahouachi M (2015). Effects of Rosmarinus officinalis L. essential oils supplementation on digestion, colostrum production of dairy ewes and lamb mortality and growth. Anim Sci J.

[40] Tassoul MD, Shaver RD (2009). Effect of a mixture of supplemental dietary plant essential oils on performance of periparturient and early lactation dairy cows. J Dairy Sci.

[41] Ceccarelli I, Lariviere WR, Fiorenzani P, Sacerdote P, Aloisi AM (2004). Effects of long-term exposure of lemon essential oil odor on behavioral, hormonal and neuronal parameters in male and female rats. Brain Res.

[42] Valente A, Carrillo AE, Tzatzarakis MN, Vakonaki E, Tsatsakis AM, Kenny GP (2015). The absorption and metabolism of a single L-menthol oral versus skin administration: effects on thermogenesis and metabolic rate. Food Chem Toxicol.

[43] Patra AK, Park T, Braun HS, Geiger S, Pieper R, Yu Z (2019). Dietary bioactive lipid compounds rich in menthol alter interactions among members of ruminal microbiota in sheep. Front Microbiol.

[44] Naumann K, Bassler R, Seibold R, Barth K (2004). Die Chemische Untersuchung Von Futtermitteln. Lose Blattausgabe Mit Ergänzungen 1983, 1988, 1993, 1997 und 2004, Methodenbuch. Chemical analysis of feed stuff, method book.

[45] NRC (2007). Nutrient requirements of small ruminants: sheep, goats, cervids, and new world camelids.

[46] Leys C, Ley C, Klein O, Bernard P, Licata L (2013). Detecting outliers: do not use standard deviation around the mean, use absolute deviation around the median. J Exp Social Psychol.

[47] SAS (2001). SAS/STAT user’s guide, version 8.

