# Supplementary material for: Dietary supplementation of menthol-rich bioactive lipid compounds alters circadian eating behaviour of sheep
Source: BMC Vet Res. 2019 Oct 21;15:352. doi: 10.1186/s12917-019-2109-0 (PMC6805686; doi:10.1186/s12917-019-2109-0)
Supplement: Supplementary file 1 — Additional file 1: Table S1. Effect of different doses of menthol-rich plant bioactive lipid compounds (PBLC) on eating time and frequency of feeder visits in different weeks of feeding. [file 12917_2019_2109_MOESM1_ESM.doc]

**Additional file 1: Table S1** Effect of different doses of menthol-rich plant bioactive lipid compounds (PBLC) on eating time and frequency of feeder visits in different weeks of feeding.

| Attribute | Treatment (Trt) | Week (wk) of study | | | Mean | SEM | *P*-value | | |
| --- | --- | --- | --- | --- | --- | --- | --- | --- | --- |
|  |  | wk 2 | wk 3 | wk 4 |  |  | Trt | wk | Trt × wk |
| Eating time (min/d) | Control | 277 | 259 | 255 | 264 | 9.73 | 0.092 | 0.17 | 0.16 |
|  | PBLC-L | 261 | 260 | 267 | 263 |  |  |  |  |
|  | PBLC-H | 295 | 292 | 284 | 290 |  |  |  |  |
|  |  |  |  |  |  |  |  |  |  |
| Feeder visit (times/d) | Control | 225 | 234 | 226 | 228^a^ | 21.7 | 0.037 | 0.055 | 0.27 |
|  | PBLC-L | 291 | 284 | 268 | 281^ab^ |  |  |  |  |
|  | PBLC-H | 321 | 285 | 258 | 288^b^ |  |  |  |  |

Sheep (*n* = 8 per treatment) were fed diets containing 0 mg/d (Control), 80 mg/d (PBLC-L) and 160 mg/d of PBLC (PBLC-H), respectively.

^a,b^Means followed by different letters within a week differ significantly (*P* < 0.05).

SEM, standard error of mean.
