# Supplementary material for: Dietary supplementation of menthol-rich bioactive lipid compounds alters circadian eating behaviour of sheep
Source: BMC Vet Res. 2019 Oct 21;15:352. doi: 10.1186/s12917-019-2109-0 (PMC6805686; doi:10.1186/s12917-019-2109-0)
Supplement: Supplementary file 2 — Additional file 2: Table S2. Effect of different doses of menthol-rich plant bioactive lipid compounds (PBLC) on the circadian distribution of eating time and feeder visits in sheep. [file 12917_2019_2109_MOESM2_ESM.doc]

**Additional file 2: Table S2** Effect of different doses of menthol-rich plant bioactive lipid compounds (PBLC) on the circadian distribution of eating time and feeder visits in sheep.

| Time-of-day (ToD) | Eating time (s/h) | | | SEM | *P*-value | | |
| --- | --- | --- | --- | --- | --- | --- | --- |
|  | Control | PBLC-L | PBLC-H |  | Trt | ToD | Trt × ToD |
| 1:00 | 15.5 | 10.2 | 47.1 | 43.0 | 0.095 | < 0.001 | 0.13 |
| 2:00 | 13.4 | 1.32 | 4.89 |  |  |  |  |
| 3:00 | 13.7 | 8.61 | 7.02 |  |  |  |  |
| 4:00 | 20.4 | 15.0 | 4.61 |  |  |  |  |
| 5:00 | 61.8 | 11.9 | 42.5 |  |  |  |  |
| 6:00 | 267 | 174 | 188 |  |  |  |  |
| 7:00 | 1726 | 1761 | 1809 |  |  |  |  |
| 8:00 | 1262 | 1582 | 1495 |  |  |  |  |
| 9:00 | 812 | 754 | 806 |  |  |  |  |
| 10:00 | 636 | 810 | 807 |  |  |  |  |
| 11:00 | 1632 | 1602 | 1751 |  |  |  |  |
| 12:00 | 361 | 357 | 467 |  |  |  |  |
| 13:00 | 380 | 434 | 440 |  |  |  |  |
| 14:00 | 790 | 499 | 560 |  |  |  |  |
| 15:00 | 2390 | 2519 | 2701 |  |  |  |  |
| 16:00 | 1129 | 1416 | 1480 |  |  |  |  |
| 17:00 | 1062 | 1132 | 1159 |  |  |  |  |
| 18:00 | 952 | 953 | 1179 |  |  |  |  |
| 19:00 | 891 | 1096 | 1096 |  |  |  |  |
| 20:00 | 633 | 697 | 802 |  |  |  |  |
| 21:00 | 208 | 111 | 252 |  |  |  |  |
| 22:00 | 25.8 | 15.9 | 49.2 |  |  |  |  |
| 23:00 | 10.1 | 5.7 | 28.8 |  |  |  |  |
| 0:00 | 26.4 | 41.7 | 14.4 |  |  |  |  |
| Time-of-day (ToD) | Feeder visits (times/h) | | | SEM | *P*-value | | |
|  | Control | PBLC-L | PBLC-H |  | Trt | ToD | Trt × ToD |
| 1:00 | 0.21 | 0.29 | 0.78 | 1.54 | 0.094 | < 0.001 | < 0.001 |
| 2:00 | 0.13 | 0.05 | 0.09 |  |  |  |  |
| 3:00 | 0.21 | 0.18 | 0.07 |  |  |  |  |
| 4:00 | 0.45 | 0.13 | 0.23 |  |  |  |  |
| 5:00 | 1.06 | 0.23 | 0.84 |  |  |  |  |
| 6:00 | 4.53 | 3.61 | 3.02 |  |  |  |  |
| 7:00 | 29.5 | 31.9 | 26.3 |  |  |  |  |
| 8:00 | 17.5^c^ | 27.4^a^ | 22.6^b^ |  |  |  |  |
| 9:00 | 11.8 | 13.2 | 12.4 |  |  |  |  |
| 10:00 | 13.3 | 14.4 | 13.2 |  |  |  |  |
| 11:00 | 24.5^y^ | 29.4^x^ | 26.6^xy^ |  |  |  |  |
| 12:00 | 4.41 | 5.09 | 6.56 |  |  |  |  |
| 13:00 | 6.53 | 7.11 | 7.11 |  |  |  |  |
| 14:00 | 12.02 | 9.38 | 10.38 |  |  |  |  |
| 15:00 | 34.7^b^ | 45.3^a^ | 36.1^b^ |  |  |  |  |
| 16:00 | 15.0^b^ | 22.6^a^ | 22.6^a^ |  |  |  |  |
| 17:00 | 13.2 | 15.8 | 17.5 |  |  |  |  |
| 18:00 | 13.4 | 14.7 | 17.1 |  |  |  |  |
| 19:00 | 11.5^b^ | 18.1^a^ | 16.7^a^ |  |  |  |  |
| 20:00 | 7.25^y^ | 10.43^xy^ | 12.09^x^ |  |  |  |  |
| 21:00 | 2.86 | 1.18 | 3.85 |  |  |  |  |
| 22:00 | 0.95 | 0.21 | 0.89 |  |  |  |  |
| 23:00 | 0.39 | 0.13 | 0.59 |  |  |  |  |
| 0:00 | 0.48 | 0.82 | 0.36 |  |  |  |  |

Sheep (*n* = 8 per treatment) were fed diets containing 0 mg/d (Control), 80 mg/d (PBLC-L) and 160 mg/d of PBLC (PBLC-H), respectively. Concentrates were fed at 07:00, 11:00 and 15:00 h of the day.

^a-c^Means followed by different superscript letters within a time point differ at *P* < 0.05.

^x-y^Means followed by different superscript letters within a time point differ at *P* < 0.10.

Trt, treatment; SEM, standard error of mean.
