# Supplementary material for: Dietary supplementation of menthol-rich bioactive lipid compounds alters circadian eating behaviour of sheep
Source: BMC Vet Res. 2019 Oct 21;15:352. doi: 10.1186/s12917-019-2109-0 (PMC6805686; doi:10.1186/s12917-019-2109-0)
Supplement: Supplementary file 3 — Additional file 3: Table S3. Effects of different doses of menthol-rich plant bioactive lipid compounds (PBLC) on eating time and frequency of feeder visits during and between feeding hours, and on body weight gain in sheep. [file 12917_2019_2109_MOESM3_ESM.doc]

**Additional file 3: Table S3** Effects of different doses of menthol-rich plant bioactive lipid compounds (PBLC) on eating time and frequency of feeder visits during and between feeding hours and on body weight gain in sheep.

|  | Time | Treatment | | | SEM | *P*-value |
| --- | --- | --- | --- | --- | --- | --- |
|  |  | Control | PBLC-L | PBLC-H |  |  |
| Eating time (min/d) | At feeding hours | 95.8 | 98.0 | 104 | 2.25 | 0.039 |
|  | Between feeding hours | 159 | 169 | 182 | 7.47 | 0.096 |
|  |  |  |  |  |  |  |
| Feeder visit (times/d) | At feeding hours | 88.6 | 107 | 89.1 | 6.55 | 0.12 |
|  | Between feeding hours | 137 | 165 | 169 | 10.5 | 0.092 |
| Body weight gain (g/d)^a^ |  | 240 | 232 | 236 | 13.1 | 0.82 |

Sheep (*n* = 8 per treatment) were fed diets containing 0 mg/d (control, CON), 80 mg/d (PBLC-L) and 160 mg/d of PBLC (PBLC-H), respectively.

SEM, standard error of mean.

^a^Body weight gain was calculated based on the final body weight at the last day of the 4^th^ week and initial body weight at first day of the 1^st^ week of the experiment.
