# Supplementary material for: Dietary supplementation of menthol-rich bioactive lipid compounds alters circadian eating behaviour of sheep
Source: BMC Vet Res. 2019 Oct 21;15:352. doi: 10.1186/s12917-019-2109-0 (PMC6805686; doi:10.1186/s12917-019-2109-0)
Supplement: Supplementary file 4 — Additional file 4: Table S4. Effect of different doses of menthol-rich plant bioactive lipid compounds (PBLC) on daily intake (g/kg body weight) of feed dry matter (DM) in different weeks. [file 12917_2019_2109_MOESM4_ESM.doc]

**Additional file 4: Table S4** Effect of different doses of menthol-rich plant bioactive lipid compounds (PBLC) on daily intake (g/kg body weight) of feed dry matter in different weeks.

| Week (wk) of study | Treatment (Trt) | | | Mean | SEM | *P*-value | | |
| --- | --- | --- | --- | --- | --- | --- | --- | --- |
|  | Control | PBLC-L | PBLC-H |  |  | Trt | wk | Trt × wk |
| wk 2 | 31.7 | 34.1 | 33.2 | 33.1^a^ | 0.72 | 0.043 | 0.022 | 0.85 |
| wk 3 | 31.3 | 33.7 | 32.5 | 32.5^ab^ |  |  |  |  |
| wk 4 | 30.6 | 33.6 | 32.2 | 32.1^b^ |  |  |  |  |

Sheep were fed diets containing 0 mg/d (Control; *n* = 8), 80 mg/d (PBLC-L; *n* = 7) and 160 mg/d of PBLC (PBLC-H; *n* = 8), respectively.

^a,b^Means followed by different letters within the same week differ significantly (*P* < 0.05).

SEM, standard error of mean.
