# Supplementary material for: Dietary supplementation of menthol-rich bioactive lipid compounds alters circadian eating behaviour of sheep
Source: BMC Vet Res. 2019 Oct 21;15:352. doi: 10.1186/s12917-019-2109-0 (PMC6805686; doi:10.1186/s12917-019-2109-0)
Supplement: Supplementary file 5 — Additional file 5: Table S5. Ingredient and chemical composition of pelleted concentrates and hay fed to sheep. [file 12917_2019_2109_MOESM5_ESM.doc]

**Additional file 5: Table S5** Ingredient and chemical composition of pelleted concentrates and hay fed to sheep.

|  | Concentrate^a^ | | | Hay |
| --- | --- | --- | --- | --- |
|  | Control | PBLC-L | PBLC-H |  |
| Ingredient composition (g/kg as-fed) |  |  |  |  |
| Corn | 305 | 295 | 285 |  |
| Barley | 305 | 305 | 305 |  |
| Soybean meal | 348 | 348 | 348 |  |
| Molasses | 30 | 30 | 30 |  |
| Mineral and vitamin premix^b^ | 5 | 5 | 5 |  |
| Salt | 2 | 2 | 2 |  |
| Limestone | 5 | 5 | 5 |  |
| PBLC premix^c^ | 0 | 10 | 20 |  |
| Chemical composition |  |  |  |  |
| Dry matter (DM; g/kg as-fed) | 914 | 915 | 912 | 923 |
| Organic matter (g/kg DM) | 949 | 950 | 948 | 958 |
| Crude protein (g/kg DM) | 259 | 257 | 259 | 108 |
| Ether extract (g/kg DM) | 30.8 | 25.1 | 25.8 | 9.60 |
| Neutral detergent fibre (g/kg DM) | 125 | 140 | 152 | 641 |
| Acid detergent fibre (g/kg DM) | 65.4 | 74.6 | 70.7 | 374 |

^a^Control: without PBLC; PBLC-L: low dose (133.3 mg/kg) of PBLC; and PBLC-H: high dose (266.7 mg/kg) of PBLC

^b^Mineral and vitamin premix (Spezialfutter Neuruppin Ltd., Neuruppin, Germany), containing per kilogram dry matter: 160 g calcium, 40 g phosphorus, 100 g sodium, 30 g magnesium, 500,000 IU vitamin A, 50,000 IU vitamin D3, 500 mg vitamin E (as alpha-tocopherol acetate), 4,500 mg zinc (as zinc oxide), 500 mg manganese (as Mn-(II)-oxide), 20 mg cobalt (as Co-(II)-carbonate), 20 mg iodine (as calcium iodate), and 35 mg selenium (as sodium selenite)

^c^PBLC premix (OAX17, PerformaNat GmbH, Berlin, Germany; containing 13.3 g PBLC/kg corn grains)
