# Supplementary material for: Dietary supplementation of menthol-rich bioactive lipid compounds alters circadian eating behaviour of sheep
Source: BMC Vet Res. 2019 Oct 21;15:352. doi: 10.1186/s12917-019-2109-0 (PMC6805686; doi:10.1186/s12917-019-2109-0)
Supplement: Supplementary file 6 — Additional file 6: Figure S1. Correlations between daily feed DM intake and daily eating time in different treatment groups. A = control, B = PBLC lower dose (80 mg/d) and C = PBLC higher dose (160 mg/d). [file 12917_2019_2109_MOESM6_ESM.ppt]

## Slide 1
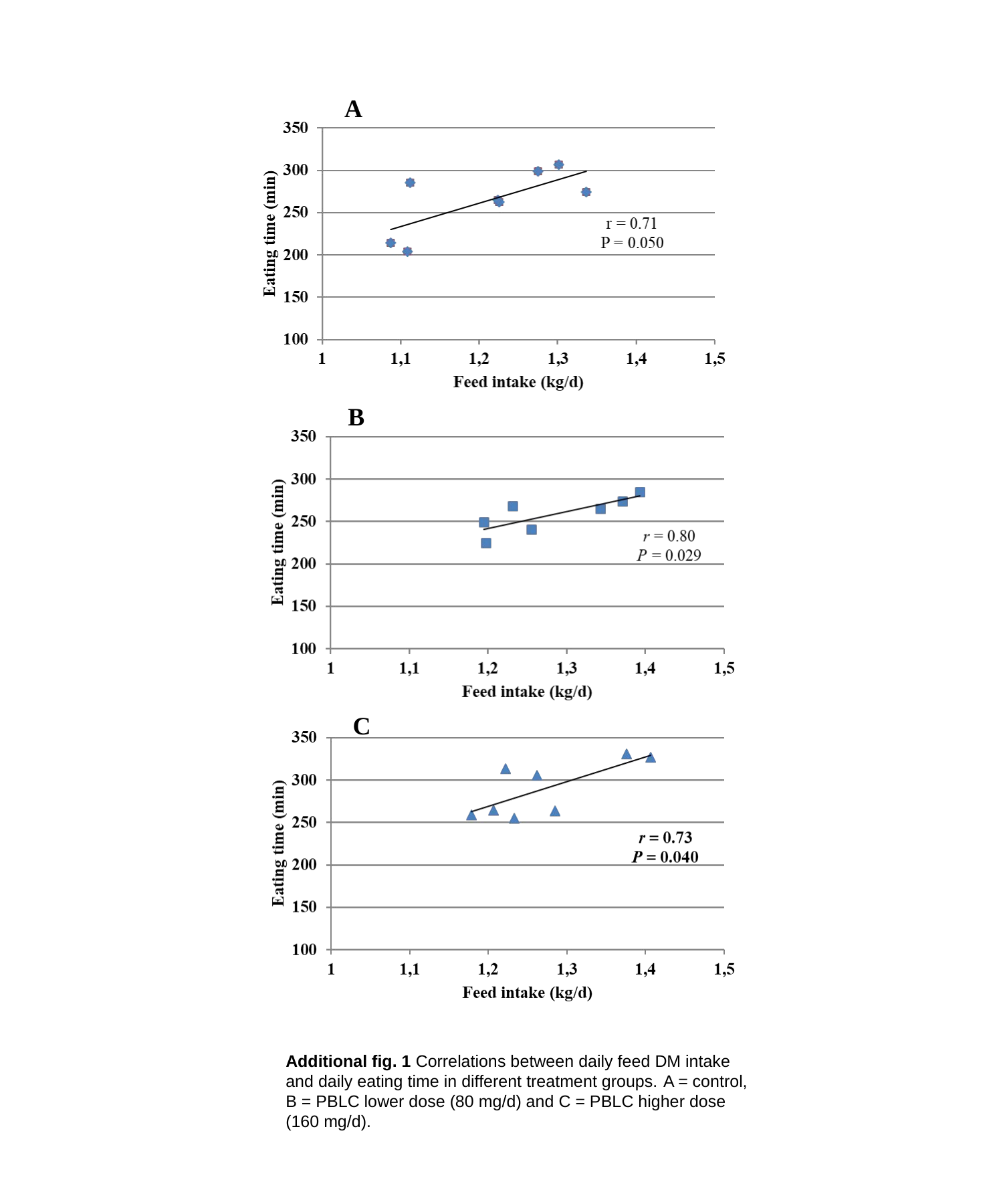

A
B
C
Additional fig. 1 Correlations between daily feed DM intake and daily eating time in different treatment groups. A = control, B = PBLC lower dose (80 mg/d) and C = PBLC higher dose (160 mg/d).
